# Effect of a natural extract of chicken combs with a high content of hyaluronic acid (Hyal-Joint^®^) on pain relief and quality of life in subjects with knee osteoarthritis: a pilot randomized double-blind placebo-controlled trial

**DOI:** 10.1186/1475-2891-7-3

**Published:** 2008-01-21

**Authors:** Douglas S Kalman, Maria Heimer, Anita Valdeon, Howard Schwartz, Eric Sheldon

**Affiliations:** 1 Departments of Nutrition and Clinical Endocrinology, Miami Research Associates, Miami, Florida 33143, USA; 2 Department of Rheumatology, Miami Research Associates, Miami, Florida 33143, USA

## Abstract

**Background:**

Intra-articular hyaluronic acid represents a substantive addition to the therapeutic armamentarium in knee osteoarthritis. We examined the effect of dietary supplementation with a natural extract of chicken combs with a high content of hyaluronic acid (60%) (Hyal-Joint^®^) (active test product, AP) on pain and quality of life in subjects with osteoarthritis of the knee.

**Methods:**

Twenty subjects aged ≥40 years with knee osteoarthritis (pain for at least 15 days in the previous month, symptoms present for ≥6 months, Kellgren/Lawrence score ≥2) participated in a randomized double-blind controlled trial. Ten subjects received AP (80 mg/day) and 10 placebo for 8 weeks. The Western Ontario and McMaster Universities Osteoarthritis Index (WOMAC) and quality of life by the Short Form-36 (SF-36v2) were administered at baseline and after 4 and 8 weeks of treatment.

**Results:**

WOMAC pain (primary efficacy variable) was similar in both study groups (mean [SD]) with 6.6 (4.0) points in the AP group and 6.4 (2.7) in the placebo group (*P *= 0.943). As compared with baseline, subjects in both groups showed statistically significant improvements in WOMAC pain, stiffness, physical function subscales, and in the aggregate score, but the magnitude of changes was higher in the AP group for WOMAC physical function (-13.1 [12.0] vs. -10.1 [8.6], *P *= 0.575) and total symptoms (-18.6 [16.8] vs. -15.8 [11.4], *P *= 0.694). At 4 weeks, statistically significant mean changes compared with baseline were observed in the SF-36v2 scales of role-physical, bodily pain, social functioning and role-emotional among subjects in the AP group, and in physical functioning, bodily pain, and social functioning in the placebo group. At 8 weeks, changes were significant for role-physical, bodily pain, and physical component summary in the AP group, and for physical functioning and role-emotional in the placebo arm. Changes in bodily pain and social functioning were of greater magnitude in subjects given AP.

**Conclusion:**

This pilot clinical trial showed that daily supplementation with oral hyaluronic acid from a natural extract of chicken combs (Hyal-Joint^®^) was useful to enhance several markers of quality of life in adults with osteoarthritis of the knee. The results warrant further study in larger sample sizes.

## Background

Osteoarthritis, the most common type of arthritis, is characterized by slow degradation of cartilage, pain, and increasing chronic disability. Osteoarthritis is a frequent cause of morbidity, functional limitations, and loss of autonomy in the second half of human life. Moreover, the disease is widely recognized to interfere with several aspects of an individual's life, including functional and social activities, body image, and psychological well-being [1]. Osteoarthritis of the knee is especially common and is a major cause of disability requiring extensive utilization of health care resources [2,3]. Medical interventions can be directed toward different stages of the disease process: patient education (e.g., weight reduction), exercise, analgesics, traditional and cyclooxygenase-2-selective nonsteroidal antiinflammatory drugs (NSAIDs), and eventually orthopedic surgery, including joint replacement. The reassessment of the central role of NSAIDs in the treatment of osteoarthritis because of suboptimal effectiveness and questions about safety [4,5] has favored the screening and development of drugs that could interfere directly with the disease process, aiming at protection and regeneration of the cartilage. Chondroprotection is a new field in the management of osteoarthritis that is designed to improve cartilage repair as well as enhance joint remodelling [6-8]. The orally administered glucosamine and chondroitin sulfate, natural components of articular cartilage, are examples of agents which may both reduce the symptoms of pain associated with osteoarthritis and have an impact on disease progression [9-12].

Hyaluronic acid or hyaluronan (sodium hyaluronate) (HA), a heteropolysaccharide comprised of a variable number of repeating units of D-glucuronic acid and N-acetylglucosamine, is synthesized by synoviocytes, fibroblasts, and chondrocytes. HA is present in the synovial fluid and the extracellular matrix of cartilage, and is responsible for the viscoelasticity and lubricating properties of synovial fluid. It has recently been shown that HA performs a multitude of biophysical, biochemical, and cell regulatory roles in joint synovial tissues [13-15]. In osteoarthritis, the concentration of synovial fluid is reduced and the viscoelastic properties of the fluid are compromised, increasing susceptibility of cartilage to injury. Intra-articular treatment with HA (viscosupplementation) has become widely accepted to reduce joint pain in osteoarthritis [16]. The original rationale for the use of intra-articular injection of HA was to increase the viscosity of the synovial fluid. The observation that the clinical result exceeded the life span of the HA exogenously administered into the joint supports the view that effects other than biomechanical properties could explain its therapeutic effectiveness. A large number of studies have been published demonstrating the positive and long lasting effects of intra-articular HA treatment in subjects with knee osteoarthritis [17-20]. A recent systematic review of 76 randomized controlled trials concluded that viscosupplementation is an effective treatment for osteoarthritis of the knee with beneficial effects on pain, function and patient global assessment [21].

These data support the use of the HA class products in the treatment of knee osteoarthritis. On the other hand, there is an increasing interest in the benefits of dietary supplements as therapeutic agents in osteoarthritis [22]. Numerous dietary supplements that may influence osteoarthritis pathophysiology, including glucosamine, chondroitin sulfate, avocado-soybean unsaponifiables, vitamins and minerals, methylsulfonylmethane, phytochemicals and plant extracts have been tested in clinical trials [23,24]. However, evidence of use of HA in oral formulations for symptoms of knee osteoarthritis is lacking. Therefore, a pilot randomized placebo-controlled trial was designed to assess the effect of dietary supplementation with a natural extract of chicken combs with a high content of hyaluronic acid (Hyal-Joint^®^) on pain relief and quality of life in subjects with osteoarthritis of the knee.

## Methods

### Study design

A randomized, double blind, placebo-controlled single-center study was designed to assess the efficacy and safety of Hyal-Joint^® ^(active test product, AP) compared to placebo for pain relief and improvement of quality of life in adult subjects with osteoarthritis of the knee. The active test product is a dietary ingredient that consists of an extract of chicken combs containing HA (60–70%) and hydrolyzed proteins mostly collagen and other polysaccharides. The duration of the study was 8 weeks. The study took place from June 2005 to April 2006 in the United States and involved the participation of a single clinical service organization center (Miami Research Associates). The study protocol was approved by the IntegReview Ethical Review Board (Austin, Texas), and was conducted in accordance with the principles of the Declaration of Helsinki and its amendments. Written informed consent was obtained from all participants prior to enrollment in the study.

### Eligibility

Male and female subjects of any race, aged 40 years or over, with symptomatic osteoarthritis of the knee were eligible provided that subjects experienced pain for at least 15 of the 30 days prior to the start of the study, symptoms had been present for at least 6 months, and a confirmatory X-ray diagnosis (Kellgren/Lawrence score ≥ 2) within the previous 6 months was available. Exclusion criteria were: known allergy to chicken (sodium hyaluronate is derived from chicken combs), corn, potato, rice or cellulose (derived from wood pulp); any inflammatory arthritic condition; multiple sclerosis or autoimmune disorder; treatment with oral corticosteroids within 4 weeks before screening; intra-articular corticosteroids in the target joint within 3 months before screening; significant injury to the target joint within the past 12 months; presence of any clinically significant medical condition judged by the investigator to preclude the patient's inclusion in the study; renal dysfunction (serum creatinine concentration > 1.5 times the upper limit of normal); liver dysfunction (serum alkaline phosphatase and aminotransferases > 2 times the upper limit of normal); and participation in a clinical study in the previous 30 days. Pregnant women, nursing mothers, or women of childbearing potential not using adequate methods of contraception were also excluded. A urine pregnancy test was performed before enrollment. New onset use of glucosamine, chondroitin sulfate, methylsulfonylmethane, or milk protein-based dietary supplements was not allowed. Subjects using these dietary products and willing to continue had to maintain stable doses within 3 months before screening and throughout the course of the study.

### Treatment and patient evaluation

Study participants attended a screening visit (visit 1), which included medical history, physical examination, laboratory tests, radiographic confirmation of Kellgren/Lawrence score ≥ 2, assessment of the use of concomitant medication and/or dietary supplements, urine pregnancy test (when applicable), and first signing the informed consent. Subjects were instructed to discontinue or taper off gradually any treatment with NSAIDs, cyclooxygenase-2 inhibitors, or analgesics with the exception of acetaminophen, to ensure a minimum 3-day drug-washout period and were scheduled to return to the study center in 7 days for the baseline/randomization visit.

All eligible participants were sequentially assigned to one of the two masked products according to a predetermined computer-generated randomization schedule. Subjects were randomized (1:1) to Hyal-Joint^® ^(Bioibérica, Barcelona, Spain), 80 mg, or matched placebo capsules. Subjects were instructed to take one capsule a day, in the morning immediately after breakfast. The study product was dispensed to subjects at each visit to cover the period of time until the next visit. On the other hand, patients were instructed to take rescue medication (paracetamol 500 mg) as needed with no more than four tablets (Tylenol) per day. The use of rescue medication was assessed by the pill return (pill count) method at each visit.

Assessments were performed at baseline (visit 2) and at 4 weeks (visit 3) and 8 weeks (visit 4) after initiation of the treatment. The Western Ontario and McMaster Universities Osteoarthritis Index (WOMAC 3.0 Index) and the Short Form-36 (SF-36v2, Acute US Version 2.0) questionnaires were administered at each visit. The WOMAC is a disease-specific, self-administered questionnaire that evaluates three dimensions: pain, stiffness, and physical function with 5, 2, and 17 questions, respectively. Each question is rated on an ordinal scale of zero to four, with lower scores indicating minimal symptoms or physical disability. The three subscales can be scored separately or as a composite measure (aggregated total symptoms). The SF-36v2 is a multi-purpose, short-form health survey with 36 questions. The SF-36v2 yields an 8-scale profile of functional health and well-being scores as well as psychometrically-based physical and mental health summary measures and a preference-based health utility index. The SF-36v2 yields information on physical health (comprised of physical functioning, role-physical, bodily pain, and general health) and mental health (comprised of vitality, social functioning, role-emotional, and mental health). Finally, the physical (PCS-36) and the mental (MCS-36) component summaries can be calculated.

### Efficacy and safety parameters

The primary efficacy variable was the comparative difference between the AP and the placebo arms in scores of the pain subscale of WOMAC and bodily pain of SF-36v2 at week 8. Secondary efficacy variables included the comparative difference between AP and placebo groups for all other WOMAC and SF-36v2 subscales, and the change over 8 weeks compared with baseline for the WOMAC and SF-36v2 subscales recorded in each study group.

At the end of the study (at 8 weeks), a subjective acceptance questionnaire was administered to determine the overall acceptability of the study products ("*Do you believe the product you were taken during the study decreased your joint pain?*" yes/no; "*Do you believe the product you were taken during this clinical trial decreased any muscle aches that you experience?" *yes/no). In addition, subjects were asked "*Do you think you were taking the active product or placebo?*".

Tolerability and safety parameters were the incidence and severity of adverse events reported throughout the study as well as changes in blood pressure, heart rate, and laboratory tests including complete blood cell count and biochemical profile. Treatment compliance was also recorded. Non-compliance was defined as taking less than 80% of the prescribed course of the study product. Use of rescue medication (paracetamol 500 mg) during the study period was also checked.

### Statistical analysis

A sample size of 20 subjects (10 per group) provided 80% power at the alpha level of 0.05 to detect a mean change over time that is about equal to the within-group standard deviations for the change over time in score points for WOMAC and SF-36v2 endpoints, which were estimated from previous studies in subjects with osteoarthritis conducted at the clinical service organization center.

All randomized subjects were included in the intent-to-treat population. The ITT population was defined as all subjects who received the product and who had some follow-up evaluation. It includes subjects who dropped out of the study, were removed or lost to follow-up, or were seriously non-compliant with the regimen specified in the protocol. The efficacy (per-protocol) population consisted of subjects who completed all visits, and who were suitably compliant with the prescribed regimen (supplement or placebo), with an overall compliance rate between 80% and 120% inclusive. Last observation carried forward (LOCF) imputation method was performed for missing efficacy variables in the intent-to-treat and per-protocol analyses (PP). The safety population included all subjects who were randomized and received at least one dose of study product and for whom any safety follow-up evaluation (physical examination, laboratory tests, adverse events, subjective comments) were obtained.

Baseline characteristics in the two study groups were compared with the Student's *t *test of the Mann-Whitney U test for continuous variables, and with the chi-square (χ^2^) test or the Fisher's exact test for categorical variables. Changes over time from baseline to each subsequent visit within each study group were assessed for significance by the paired Student's *t *test or the non-parametric Wilcoxon signed-rank test. Statistical significance was set at *P *< 0.05.

## Results

### Study population

Of the 36 potential participants phone-screened, 16 were not enrolled because of lack of eligibility at the screening visit (*n *= 15) and lost to follow-up before the baseline/randomization visit (*n *= 1). Therefore, the study population included 20 subjects, 9 men and 11 women, with a mean (SD) age of 56.3 (9.0) years (range 42–73 years). A total of 11 subjects were randomized to the active supplement and 9 to placebo. However, three subjects in the AP arm discontinued the study because of personal or professional reasons unrelated to the study. One subject in the placebo arm discontinued the study due to restrictions imposed by his work schedule. Data of the remaining 16 evaluable subjects were included in the PP data set. At baseline, there were no significant differences in demographic data, anthropometric measures, vital signs, and scores of the WOMAC and SF-36v2 instruments between subjects assigned to the active supplement and those assigned to placebo (Table 1). Results of laboratory tests were within normal ranges in both study groups.

**Table 1:** Baseline characteristics of the study subjects

Variable	Study group		*P *value
	Hyal-Joint^® ^(*n *= 11)	Placebo (*n *= 9)	
Men/women	4/7	5/4	0.653
Age, years, mean (SD)	57.7 (10.1)	54.6 (7.7)	0.448
Ethnicity			0.361
Black	0	1	
Caucasian	1	1	
Hispanic	10	6	
Height, cm, mean (SD)	164.7 (6.1)	164.1 (10.8)	0.889
Weight, kg, mean (SD	79.1 (26.3)	80.9 (20.7)	0.867
Heart rate, beats/min, mean (SD)	75.5 (12.3)	75.7 (12.4)	0.970
Systolic BP, mm Hg, mean (SD)	122.8 (16.4)	135.6 (21.7)	0.152
Diastolic BP, mm Hg, mean (SD)	77.8 (8.9)	84.3 (10.4)	0.148
Rescue medication use (paracetamol 500 mg)*			
No. capsules (range)	4 (0–19)	9 (0–29)	0.425
Mean (SD)	6.8 (7.3)	9.9 (9.6)	
WOMAC scores, mean (SD)			
Pain	10.4 (3.0)	10.1 (2.7)	0.848
Stiffness	4.4 (1.0)	4.4 (1.0)	0.862
Physical function	37.7 (7.4)	35.4 (11.5)	0.597
Aggregated total symptoms	52.5 (10.8)	50.0 (14.4)	0.667
SF-36v2 scores, mean (SD)			
Physical functioning	30.8 (8.5)	27.1 (8.5)	0.342
Role-physical	35.3 (8.3)	41.9 (7.8)	0.084
Bodily pain	33.2 (4.8)	36.9 (4.8)	0.100
General health	44.1 (12.3)	45.4 (10.3)	0.814
Vitality	49.0 (11.3)	50.4 (11.7)	0.791
Social functioning	37.5 (12.0)	43.5 (11.3)	0.268
Role-emotional	36.1 (12.6)	40.3 (12.9)	0.467
Mental health	40.3 (19.7)	47.5 (9.5)	0.326
Physical component summary (PCS-36)	34.8 (6.3)	34.6 (6.4)	0.947
Mental component summary (MCS-36)	43.6 (17.5)	50.6 (11.2)	0.317

*Between screening and randomization visits.

### Efficacy

With regard to results of the WOMAC Index, the comparison of mean (SD) scores for the pain dimension between the study groups (primary efficacy variable) did not show statistically significant differences (AP 6.3 [4.0]; placebo 6.4 [2.7], *P *= 0.943). However, within-group differences for pain, WOMAC physical function, stiffness, and aggregated total symptoms score as compared with baseline were statistically significant at any (or both) of the follow-up visits. On the other hand, the magnitude of mean (SD) changes from baseline to week 8 was higher for subjects treated with AP compared with placebo for WOMAC physical function (-13.1 [12.0] *vs *-10.1 [8.6]) and total symptoms (-18.6 [16.8] *vs *-15.8 [11.4]), although these between-group differences were not statistically significant. However, changes from baseline to week 4 for physical function (-8.1 [6.3], *P *= 0.008) and total symptoms score (-10.1 [9.6], *P *= 0.20) achieved statistical significance only in the AP group. Detailed results of the WOMAC Index are shown in Table 2.

**Table 2:** WOMAC Index scores during the study period in 16 evaluable subjects

Study groups and time points	WOMAC Index scores, mean (SD)			
	
	Pain	Stiffness	Physical function	Total symptoms
Hyal-Joint^® ^(*n *= 8)				
Baseline	10.4 (3.6)	4.2 (1.0)	36.3 (7.7)	50.9 (12.0)
Week 4	8.8 (3.4)	3.9 (1.6)	28.1 (9.2)	40.8 (13.6)
Week 8	6.3 (4.0)	2.9 (1.9)	23.1 (15.1)	32.3 (20.8)
Change baseline *vs *week 4	-1.6 (2.7)	-0.4 (1.5)	-8.1 (6.3)	-10.1 (9.6)
*P *value	0.135	0.504	**0.008**	**0.020**
Change baseline *vs *week 8	-4.1 (3.4)	-1.4 (2.0)	-13.1 (12.0)	-18.6 (16.8)
*P *value	**0.012**	0.092	**0.018**	**0.016**
Placebo (*n *= 8)				
Baseline	10.4 (2.7)	4.5 (1.9)	37.4 (10.6)	52.3 (13.6)
Week 4	8.3 (3.0)	3.5 (1.9)	30.4 (10.7)	42.1 (17.7)
Week 8	6.4 (2.7)	2.9 (1.0)	27.3 (10.7)	36.5 (13.7)
Change baseline *vs *week 4	-2.1 (3.3)	-1.0 (2.1)	-7.0 (10.8)	-10.1 (15.3)
*P *value	0.113	0.227	0.110	0.103
Change baseline *vs *week 8	-4.0 (2.8)	-1.6 (0.9)	-10.1 (8.6)	-15.8 (11.4)
*P *value	**0.005**	**0.002**	**0.012**	**0.006**

**Figure 1:**
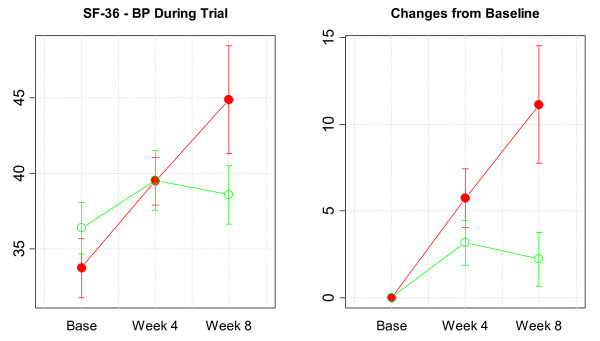
Average change from baseline in SF-36v2 bodily pain score during the trial (higher is better) (solid symbols = active product, open symbols = placebo).

**Figure 2:**
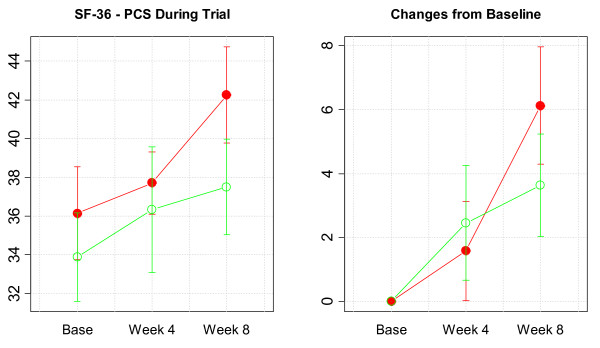
Average change from baseline in SF-36v2 physical component summary score during the trial (higher is better) ((solid symbols = active product, open symbols = placebo).

For the primary efficacy endpoint of the SF-36v2 (bodily pain), differences between the study groups at week 8 were not statistically significant (AP 44.9 [10.1]; placebo 38.6 [5.4], *P *= 0.143), although subjects in the active supplement group scored higher than those given placebo. Within-group changes for bodily pain from baseline to week 4 were statistically significant in both study groups; however, changes from baseline to week 8 were only statistically significant in the AP arm (11.1 [9.6], *P *= 0.013) (Figure 1). Individualized results of dimensions of physical health and mental health of the SF-36v2 questionnaire are detailed in Tables 3 and 4, respectively. With regard to PCS-36 (Figure 2), mean scores in the AP arm improved significantly from 36.1 (6.8) at baseline to 42.3 (7.0) at the end of the study (mean change 6.1 [5.2], *P *= 0.012), whereas in subjects given placebo scores increased from 33.9 (6.5) at baseline to 37.5 (6.9) at week 8 (mean change 3.6 [4.5], *P *= 0.059). Neither within-group nor between-group differences for MCS-36 were observed.

**Table 3:** Results of the physical health dimension of the SF-36v2 in 16 evaluable subjects

Study groups and time points	SF-36v2 scores, mean (SD)			
	
	Physical functioning	Role-physical	Bodily pain	General health
Hyal-Joint^® ^(*n *= 8)				
Baseline	31.8 (9.5)	36.1 (9.3)	33.7 (5.5)	48.5 (9.9)
Week 4	34.9 (7.1)	42.2 (7.2)	39.5 (4.5)	46.0 (10.8)
Week 8	34.2 (11.0)	42.8 (6.8)	44.9 (10.1)	51.3 (9.2)
Change baseline *vs *week 4	3.1 (5.2)	6.1 (3.9)	5.8 (4.7)	-2.5 (8.8)
*P *value	0.137	**0.003**	**0.011**	0.444
Change baseline *vs *week 8	2.4 (5.2)	6.8 (7.8)	11.1 (9.6)	2.8 (9.7)
*P *value	0.239	**0.045**	**0.013**	0.439
Placebo (*n *= 8)				
Baseline	25.5 (7.5)	41.6 (8.3)	36.4 (4.8)	46.5 (10.4)
Week 4	30.5 (9.7)	41.9 (11.6)	39.5 (5.6)	44.4 (8.4)
Week 8	33.1 (9.0)	44.6 (9.3)	38.6 (5.4)	48.4 (7.9)
Change baseline *vs *week 4	5.0 (5.1)	0.3 (5.6)	3.2 (3.7)	-2.1 (4.9)
*P *value	**0.028**	0.884	**0.045**	0.267
Change baseline *vs *week 8	7.6 (5.2)	3.1 (5.0)	2.2 (4.4)	1.9 (5.5)
*P *value	**0.004**	0.129	0.199	0.360

**Table 4:** Results of the mental health dimension of the SF-36v2 in 16 evaluable subjects

Study groups and time points	SF-36v2 scores, mean (SD)			
	
	Vitality	Social functioning	Role-emotional	Mental health
Hyal-Joint^® ^(*n *= 8)				
Baseline	49.4 (9.9)	38.4 (10.1)	36.9 (10.9)	42.3 (18.8)
Week 4	50.9 (4.4)	46.6 (8.5)	44.2 (5.5)	49.7 (13.3)
Week 8	52.1 (4.7)	43.2 (12.4)	42.3 (10.2)	44.4 (16.3)
Change baseline *vs *week 4	1.6 (9.9)	8.2 (8.8)	7.3 (7.3)	7.4 (12.2)
*P *value	0.669	**0.033**	**0.026**	0.131
Change baseline *vs *week 8	2.7 (11.9)	4.8 (12.5)	5.4 (8.8)	2.1 (15.2)
*P *value	0.537	0.318	0.129	0.707
Placebo (*n *= 8)				
Baseline	49.8 (12.3)	44.6 (11.6)	39.4 (13.4)	48.3 (9.9)
Week 4	49.4 (9.1)	49.3 (8.2)	38.9 (11.6)	49.7 (10.9)
Week 8	53.3 (6.5)	46.6 (8.9)	44.7 (9.6)	52.5 (8.4)
Change baseline *vs *week 4	-0.4 (8.4)	4.8 (4.5)	-0.5 (7.0)	1.4 (9.4)
*P *value	0.897	**0.021**	0.843	0.684
Change baseline *vs *week 8	3.5 (9.3)	2.0 (7.7	5.3 (6.2)	4.2 (9.7)
*P *value	0.323	0.477	**0.045**	0.258

The mean (SD) number of capsules of acetaminophen used per week was higher among subjects assigned to placebo (4.3 [3.6]) than among those using the active supplement (2.0 [3.0]) (*P *= 0.184), although this was not significant. Use of rescue medication during the course of the study is shown in Table 5. Additionally, more subjects in the AP group compared with placebo answered affirmatively to perceived improvement in joint pain (75% *vs *50%) and muscle aches (75% *vs *38%). Sixty-two percent of subjects in the active supplement group thought they had received AP and 50% in the placebo group thought they were in the comparator arm.

**Table 5:** Usage of rescue medication (acetaminophen 500 mg) during the study

Data	Study group		*P *value
		
	Hyal-Joint^® ^(*n *= 11)	Placebo (*n *= 9)	
Subjects using rescue medication			
Randomization to week 4, *n *= 12	6	6	1.00
Week 4 to week 8, *n *= 12	6	6	1.00
Usage during the first 4 weeks			
Mean number of capsules (SD)	3.1 (4.2)	6.8 (6.0)	0.172
Capsules per week (range)	1.2 (0 – 12.1)	7 (0 – 16.5)	
Usage during the study			
Mean number of capsules (SD)	2.0 (3.0)	4.3 (3.6)	0.184
Capsules per week (range)	1.1 (-0.7 – 8.5)	3.6 (0 – 9.5)	

### Safety and tolerability

Three adverse events were observed during the study period. One subject in the AP group complained of acute non-target knee pain, unrelated to the study product, and voluntarily dropped out of the study. The two adverse events among placebo subjects, one diarrhea episode and one hypoesthesia of the tongue, were of mild intensity and were judged by the investigator as probably not related (diarrhea) and possibly related (hypoesthesia) to the study product. No significant changes were observed in vital signs, body weight, and results of laboratory tests. Compliance with the study product was above 90% in both groups at each time point, with an overall compliance rate of 97%.

## Discussion

In this pilot randomized, double-blind, controlled trial, orally delivered HA in the form of a dietary supplement appeared to have some efficacy for pain relief in subjects with osteoarthritis of the knee as shown by within-group statistically significant changes from baseline to the final visit in the mean scores for pain subscale of WOMAC Index and bodily pain subscale of SF-36v2. On the other hand, scores of the PCS-36 also improved significantly in the active supplement group.

Although significant within-group differences in the pain subscales at weeks 4 or 8 compared with baseline were also observed among subjects given placebo, the magnitude of pain relief was higher in the AP arm. With regard to improvement in health-related quality of life, the active supplement was also more favorable than placebo in promoting early improvements in the physical function subscale and aggregated total symptoms score of WOMAC, as shown by within-group statistically significant differences at 4 weeks. In the role-physical subscale of SF-36v2, within-group differences at 4 and 8 weeks time points were only statistically significant in the active supplement arm. Moreover, the percentage of subjects reporting pain relief in the AP group was 75% compared to 50% in the placebo arm. Other randomized, double-blind, placebo-controlled studies carried out in patients with osteoarthritis of the knee have shown a beneficial effect of nutrition supplementation with joint specific substances on pain reduction. After 4 weeks of supplementation with 1500 mg/day of glucosamine sulfate, 52% of patients in the study group reported an improvement in pain assessed with the Lequesne's index, while in the placebo group the percentage of responders was only 37% [25]. Comparable results were obtained after supplementation with avocado/soybean with 53% of the patients in the study group judging their treatments as effective compared to only 30% in the placebo group [26]. When the effects of a ginger extract on knee pain in patients with osteoarthritis was assessed, the percentage of responders experiencing a reduction in knee pain on standing was 63% in the ginger extract group versus 50% in the control group [27]. According to these data, the higher magnitude of pain relief observed in the AP group can be considered meaningful. The preliminary study described herein provides some evidence, although preliminary, that oral HA is efficacious for reducing pain and subsequently improving quality of life in adult with knee osteoarthritis.

More subjects in the placebo group (6/9) than in the active product group (6/11) used rescue medication to some extent over the course of the study. The placebo group tended to use twice as much acetaminophen capsules as the AP group, but the difference was not statistically significant, which may be attributable to the small number of subjects in this pilot study.

The present findings, however, should be interpreted taking into account the limitations of the study, particularly the small study population given the exploratory and pilot characteristics of the trial and the fact that the duration of the study was limited to 8 weeks. Moreover, the effects of HA supplementation on joint structure morphology were not assessed. Experimental studies have shown a stimulatory effect of HA on chondrocyte metabolism [28-31]. These stimulating effects could lead to a permanent improvement on the cartilage if the concentration of HA could be elevated constantly for a longer period of time. One possibility to meet a higher demand caused by a physiological dysfunction is usually the oral administration of specific nutrients or combinations. Long-term studies are needed to investigate possible structural effects of the substance. A further advantage of supplementing patients with joint-specific nutrients is the long-term efficacy. The reduction of pain associated with use of NSAIDs in osteoarthritis disappears quickly when medication is discontinued. Supplementation with joint-specific nutrients has been shown to produce similar effects on pain after longer intake, but with persistent improvements after the end of supplementation [26,32,33].

Results of this study cannot be compared with data reported by others. As far as we are aware, no previous study has examined the clinical impact of oral HA supplementation in subjects with osteoarthritis of the knee. However, the results appear to build on with prior studies on intra-articular HA (i.e., Synvisc^®^, Hyalgan^®^). Considering the risks associated with many of the analgesic and antiinflammatory medications used to treat a chronic condition, such as osteoarthritis, and side effects associated with the long-term use of NSAIDs, it is appropriate to consider the adjunctive use of HA in the therapeutic armamentarium of knee osteoarthritis. This nutritional product may be particularly advantageous for elderly subjects in which osteoarthritis-related pain is a serious limitation of activities of daily living. Oral HA supplementation may also offer a benefit over intra-articular HA avoiding potential complications at the injection site and discomfort associated with repeated injections [18].

## Conclusion

Daily supplementation with HA from a natural extract of chicken combs (Hyal-Joint^®^), 80 mg/day for 8 weeks, appeared to be effective in subjects with knee osteoarthritis for decreasing pain, improving physical function, and enhancing several aspects of quality of life. Results of this pilot trial warrant further study in a larger population.

## Abbreviations

CI: Confidence interval.

HA: Hyaluronic acid.

NSAIDs: Nonsteroidal antiinflammatory drugs.

LOCF: Last observation carried forward.

MCS-36: Mental component summary-36.

PCS-36: Physical component summary-36.

PP: Per-protocol.

SF-36v2: Short Form-36 Acute US Version 2.

SD: Standard deviation.

WOMAC: Western Ontario and McMaster Universities Osteoarthritis Index.

## Competing interests

The author(s) declare that they have no competing interests.

## Authors' contributions

Douglas S. Kalman, MS, RD, FACN, designed the study, reviewed data, and interpreted results.

Maria Heimer, CCRC, coordinated the study and collected data.

Anita Valdeon BPS, CCRC, coordinated the study and collected data.

Howard I. Schwartz, MD, FACP, was the sub-investigator and designed the study.

Eric Sheldon, MD, FACR, was the principal investigator and designed the study.

All authors reviewed the manuscript and approved the final draft.
